# Enhancing and assessing fidelity in the TANDEM (Tailored intervention for ANxiety and DEpression Management in COPD) trial: development of methods and recommendations for research design

**DOI:** 10.1186/s12874-022-01642-5

**Published:** 2022-06-06

**Authors:** Steed L., Wileman V., Sohanpal R., Kelly MJ., Pinnock H., Taylor SJC

**Affiliations:** 1 Wolfson Institute of Population Health, Barts and the London School of Medicine and Dentistry, Queen Mary University of London, 58 Turner Street, London, E1 2AB UK; 2 Allergy and Respiratory Research Group, Usher Institute, Doorway 3, Medical School, Teviot Place, Edinburgh, EH8 9AG UK

**Keywords:** Fidelity, Methodology, Therapeutic Competence, Assessment, COPD, Depression, Anxiety, Complex Intervention

## Abstract

**Background:**

Development of complex interventions for management of chronic conditions has become increasingly common, with guidance now provided. Fidelity (whether the intervention is designed, delivered and received as intended) is critical to understanding if, and how an intervention works (or not). However, methods for achieving this are still evolving. This study describes the methods used in the TANDEM trial – a large multicentre study evaluating the impact of a cognitive behavioural intervention preceding routine pulmonary rehabilitation for people with chronic obstructive pulmonary disease and anxiety and or depression. Results for enhancement and training aspects of fidelity, are presented.

**Methods:**

Using the National Institute for Health Behaviour Change Consortium (NIH BCC) framework of fidelity, a set of enhancement strategies and a fidelity measurement strategy were developed with input from a multidisciplinary team. The Cognitive First Aid Rating Scale (CFARS) was used to assess Facilitator (the respiratory professional delivering TANDEM) therapeutic competence at the end of the initial training and throughout treatment delivery (on a randomly selected set of cases). A TANDEM specific treatment adherence measure was developed following previously recommended procedures. Together these (the CFARS and adherence measure) comprised the TANDEM treatment delivery fidelity tool.

**Results:**

Hiring of respiratory professionals to the initial training programme was successful, with 44% of those expressing initial interest in being a Facilitator successfully completing the process. Video recordings of potential Facilitators conducting standardized patient role plays at the end of the initial training demonstrated fidelity of training.

**Conclusions:**

Addressing fidelity in complex intervention trials is a time and resource intensive process but has significant potential to increase understanding of results and strengthen the evidence base for effective interventions. By defining a full fidelity assessment method *prior to analysis* we aimed to minimise bias when interpreting results.

**Trial registration:**

ISRCTN59537391. Registered on 20 March 2017. Trial protocol version 6.0, 22 April 2018. Process evaluation protocol version 4.0, 1 November 2020.

**Supplementary Information:**

The online version contains supplementary material available at 10.1186/s12874-022-01642-5.

## Background

Chronic Obstructive Pulmonary Disease (COPD) is one of the leading causes of morbidity and mortality worldwide with a global prevalence rate of 11.7% in adults over 30 years of age [1, 2]. The progressive, irreversible deterioration in lung function reduces physical capacity, quality of life and life expectancy [3]. Characterised by the fear-invoking symptom of breathlessness it is not surprising that levels of anxiety and depression are also high in this population [4–8] with a cited prevalence for anxiety of 10–50% [5, 7] and approximately 30% for depression [4, 7]. Patients can get caught in vicious cycles of fear avoidance and emotional decline, which further exacerbates their condition [9].

Evidence-based strategies for managing both COPD and the psychological comorbidities of anxiety and depression have been established. Pulmonary Rehabilitation (PR) is a guideline recommended treatment [10] that improves functional capacity, psychological well-being, and quality of life in COPD [10–12]. Similarly Cognitive Behavioural Therapy (CBT) has shown promise for managing anxiety and depression including in COPD [9, 13–15]. It has been hypothesised that the integration of CBT and PR could benefit patients with co-morbidity [11] through reducing mood disorders and increasing attendance and completion of PR, with potential synergistic effects. A large multi-site trial (TANDEM) has been conducted to test this hypothesis [16] and examine whether an intervention with a cognitive behavioural approach (CBA) which precedes, links into, and optimises PR uptake, can improve outcomes for people with moderate to very severe COPD and co-morbid mild to moderate anxiety and /or depression. The trial was funded by the NIHR Health Technology Assessment programme and was designed in response to a commissioned call for research in this area.

The study protocol for TANDEM has been published [16] and describes a pragmatic multi-centre randomized controlled trial (*n* = 423) comparing an intervention group to an usual care control group. Eligible participants were those with moderate to very severe COPD and mild to moderate anxiety and/ or depression, who were eligible for pulmonary rehabilitation and could be recruited from primary, secondary or community care. Participants were recruited prior to pulmonary rehabilitation and offered a 6–8 week tailored, one-to-one, face-to-face CBA intervention that also included promotion of self-management skills and was linked to subsequent PR. The intervention development paper [17] describes the full programme theory (underpinned by cognitive behaviour theory [18] and self-regulatory theory [19]), but in brief this hypothesised that individuals with anxiety and or depression would be less likely to attend pulmonary rehabilitation, due to a range of mechanisms such as fear of breathlessness, withdrawal from activity and decreased self-management ability. By targeting these mechanisms TANDEM proposed to improve mood symptoms, increase uptake of PR and lead to a synergistic improvement in outcomes such as symptoms of anxiety and depression (co-primary outcomes), health care utilization and cost-effectiveness. A three day initial training course for respiratory professionals who delivered the intervention (‘TANDEM Facilitators’) was mandatory and delivered by experienced respiratory and health and clinical psychology trainers. Throughout delivery of the intervention TANDEM Facilitators were required to undergo individual supervision sessions of approximately thirty minutes on a fortnightly basis by a senior qualified practitioner in CBT which contributed to further training and skill development.

Informed by the UK Medical Research Council recommendations for conduct of process evaluations [20] the TANDEM comprehensive process evaluation [21] included assessment of intervention fidelity. Fidelity has been defined as ‘the ongoing assessment, monitoring and enhancement of the reliability and internal validity of a study’ [22] and is commonly described as ‘whether an intervention and study is delivered as planned’ [23]. There are two common components to fidelity [22] firstly treatment integrity, the degree to which the intervention is delivered as intended (including both intervention and control arms) and secondly treatment differentiation, the extent that different arms of the study consistently differ. The plethora of publications in the area in recent years is testament to the increasing importance attached to ensuring and assessing fidelity [24]. Recommendations for how to assess [25] and report fidelity [26] have been published and build upon the early seminal work in the area by the National Institute of Health Behaviour Change Consortium (NIH BCC) [27, 28] which described five key domains of fidelity: treatment design, training providers, delivery of treatment, receipt of treatment, enactment of treatment. Toomey et al. [26] go further by arguing that strategies to enhance fidelity should be considered at the design and development stage of an intervention.

**Fig. 1:**
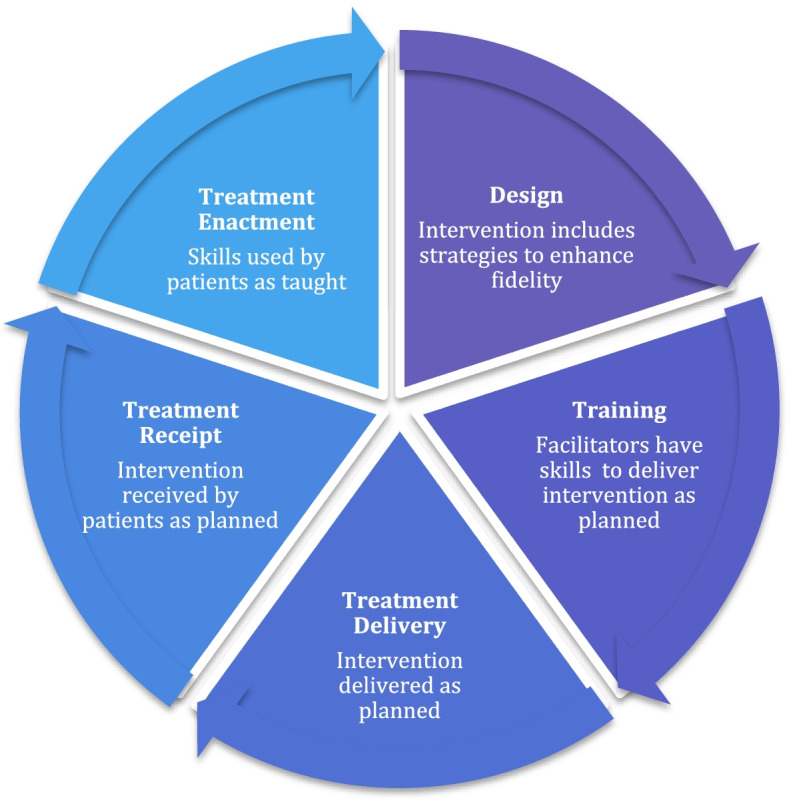
Proposed Process of Fidelity incorporating NIH BCC fidelity domains [25, 26]

In this paper we report our methods for enhancing fidelity and developing a method for assessing fidelity in TANDEM. All five domains of fidelity are considered but with particular emphasis on how we assessed the fidelity of initial training of Facilitators and fidelity of treatment delivery, as these elements cannot be inferred from other trial outcomes. In developing our fidelity strategy, we were conscious that high fidelity of latter domains such as ‘treatment delivery’ were incumbent on successful fidelity in earlier domains such as ‘initial training’ thus we conceptualised fidelity as a process (see Fig. 1) rather than mutually exclusive domains. We therefore present the results of fidelity to initial training in this publication, whilst other fidelity results will be reported upon completion of the trial and process evaluation. We also present lessons learnt and recommendations for future research. Finally, we argue that designing a fidelity method prior to trial analysis (effectively a protocol for fidelity), as with other aspects of trial design, is essential in order to minimise bias and ensure transparency.

## Methods

### Conceptualisation of fidelity and guiding framework

To guide our methodology we used the NIH BCC framework [27, 28] and were guided by the work of Toomey et al. [26]. We therefore aimed to consider how to both enhance and assess fidelity for each of the five NIH BCC domains. As described above we conceptualised fidelity as a process, such that fidelity at earlier phases was assumed to impact on fidelity at latter stages.

**Fig. 2:**
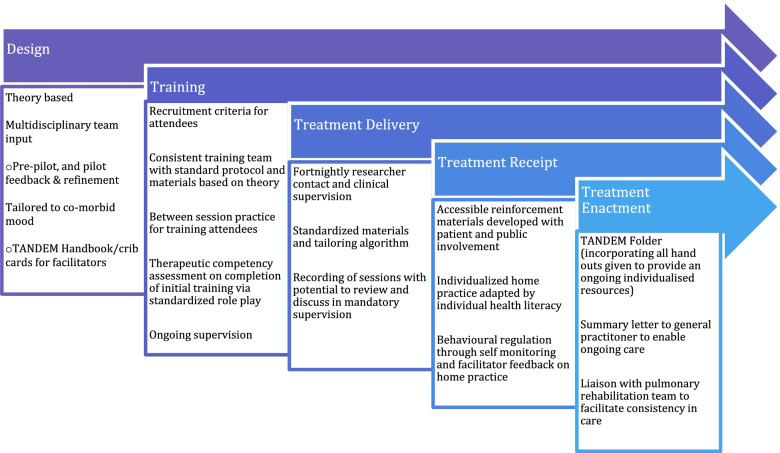
Enhancing Fidelity in TANDEM

Borelli (2011) [22] has suggested comprehensive strategies to enhance fidelity within each of the five domains (see Fig. 1). We used these from the outset (in developing our grant application) as a guide for intervention development. An iterative process was undertaken by the lead author supported by a multidisciplinary team (consisting of health psychologists, academic general practitioners, public health professionals, qualitative researchers and health service researchers) whereby the evolving intervention was reviewed and refined in light of recommendations. The intervention was reviewed by the team post piloting to consider if further enhancement of fidelity was possible. Figure 2 illustrates the key enhancement strategies under each of the five fidelity domains and supplementary table 1 provides a full description of strategies and links these to recommendations by Borrelli et al. [22] of how fidelity should be enhanced.

In order to standardise delivery, and enhance fidelity in the control group, all control participants were provided with the same consistent educational information in addition to receiving a referral for PR assessment. This comprised the British Lung Foundation (BLF) publicly available DVD: ‘Living with COPD’/ ‘Stay Well Stay Active’ and BLF COPD information on exercise and a PR booklet which provides advice in accordance with national guidelines [29] to facilitate the control group receiving best standard care.

To target treatment differentiation, as mentioned above the second component of fidelity [22], and to avoid treatment burden, the trial design specifically excluded individuals who were currently receiving a psychological intervention, or who had received one within the preceding six months.

### Assessment of fidelity domains

#### Design

To monitor intervention and assessment delivery and detect protocol deviations a case report form was developed in line with SPIRIT recommendations [30] and has been reported previously [16]. In brief this form collected when each activity was conducted, by whom, and the duration of the task. In this way any deviations from protocol were detected.

#### Training

##### Hiring facilitators

A job specification and description for the Facilitator role was developed. The role was advertised through social media (i.e., Twitter) and respiratory networks such as the Association of Respiratory Nurse Specialists, and at events such as the Primary Care Respiratory Society annual conference. Interested professionals were invited to submit a curriculum vitae and a personal statement to the study team, and those fulfilling criteria (e.g. registered respiratory professional; able to commit a day a week to TANDEM) were invited to a structured telephone interview. The interview was conducted by one of the chief investigators with a second interview with a health psychologist if deemed necessary. All potential Facilitators had to demonstrate a commitment to a biopsychosocial approach to treatment, willingness to travel and readiness to see participants in their homes before a place on the training programme was offered. A study log was kept of the full recruitment process.

##### Training facilitators

A standardised initial training programme was developed with slide sets, demonstration videos and specified exercises for use by all trainers. All training sessions were video recorded to enable comparison with the protocol if resources allowed. In addition, as skills often develop and must be maintained over time, we required all Facilitators to have regular individual supervision of approximately thirty minutes every fortnight. It was possible for Facilitators to use their audio-recordings of sessions if desired, although this was not a specific requirement of supervision. The number of planned supervision sessions received was measured to indicate dosage of supervision received.

In order to assess Facilitator therapeutic competence these post-initial training, all Facilitators underwent an individual face-to-face, video-recorded assessment with a trained actor playing the role of a patient. The task was to conduct an initial formulation, using a cognitive behavioural approach as taught in the training, and present this back to the patient. The same actor was used for all assessments and they received training on the scenario to be acted before delivery. Use of standardized role plays in training for cognitive behavioural approaches has been recommended previously [31, 32].

The video-recording of each Facilitator was independently coded by two psychologists (LS & VW) using the Cognitive First Aid Rating Scale (CFARS) [33]. The CFARS is a 10 item scale based upon the Revised Cognitive Therapy Rating Scale (CTS-R) [34] but adapted to be more appropriate for health practitioners who are delivering a cognitive behavioural approach, rather than full cognitive behavioural therapy. The CFARS was developed, and has demonstrated reliability and validity, in the context of palliative care practitioners who had received a brief training course on cognitive behavioural skills [33]. This was judged appropriate to TANDEM which provided brief training for a cognitive behavioural approach with a population with physical health difficulties, in line with the previously defined competencies [35, 36]; in addition the CFARS had been used with a COPD population [14, 15].

For each of the 10 items on the CFARS there is a 7-point scale (0 = incompetent, to 6 = expert) giving a 60-point total. We omitted item 9 ‘application of appropriate change techniques’ from our assessment scoring as we did not require Facilitators to show application of change techniques within the evaluated role-play. In line with previous guidance we required a minimum total score of 50% (raw score 27) and no item falling below 2 as an indication of sufficient competence [33]. Where there was disagreement between coders which could not be resolved through discussion typically the mean score was taken, or if necessary, a third coder (SCT) arbitrated.

All Facilitators received one to one, face to face, feedback on their assessment videos to enhance their therapeutic competence and fidelity to treatment delivery [37].

#### Treatment delivery

All sessions delivered by TANDEM Facilitators were audio-recorded (with participant permission) and a random sample of full cases (i.e. all sessions delivered to that participant) coded for fidelity of treatment delivery. If Facilitators delivered the intervention to nine or fewer participants one full case was randomly selected from their first five cases for review. If Facilitators delivered to ten or more participants then two full cases were reviewed (one randomly selected from their first five cases and a second randomly selected from their 10-15th participant to allow for possible change over time to be seen). Overall, we intended to assess 10% of cases.

All of the sessions within the randomly selected cases were coded by a psychologist (VR) trained in behavioural interventions who was independent of the study team. Twenty percent of these were second coded by a member of the study team (LS) to ensure quality of the main coder; however, to ensure consistency, the scores of the primary coder were used for analysis.

Both therapeutic competence and treatment adherence should be assessed in the fidelity evaluation of treatment delivery [23]. In order to assess therapeutic competence the full 10-item CFARS was used and treatment adherence was measured by a TANDEM specific treatment adherence scale.

#### Development of the TANDEM Specific treatment adherence scale

A review of the literature did not identify a suitable validated tool for assessment of adherence to treatment delivery. This was expected as TANDEM is a novel and tailored intervention, hence it was necessary to create a bespoke checklist which was developed in line with recommendations by Walton et al. [25] which describes a 5-step process involving reviewing previous measures (as noted above this did not identify a suitable measure), analysing the intervention, developing a coding system, checking wording with the team and piloting.

Three individuals (LS, VW, ST) completed a comprehensive analysis of the TANDEM Facilitators Handbook (study manual) and all other patient materials to identify the core and topic specific elements of the delivered TANDEM intervention. Thus, an initial coding framework was developed. An attempt was made at this stage to code each task according to the Michie Behaviour Change Taxonomy v1 [38], however this was not successful given insufficient cognitive techniques (e.g. challenge thoughts) in the taxonomy.

In understanding adherence there is a further distinction that needs to be recognised, that between behaviours/tasks that should be delivered, and content/information that should be delivered. As TANDEM is a tailored intervention, certain behaviours (which we describe as core tasks) were required every session, however the content i.e. topic this related to, was dependent on the needs of the individual (see Steed et al. [17] for further discussion on tailoring within the TANDEM intervention). The TANDEM specific treatment adherence scale was therefore split into two sections i) for core tasks that were required repeatedly e.g., set agenda, discuss homework, refer to hot cross bun (this is a CBT term referring to a formulation based on how thoughts, feelings, behaviours and symptoms inter-relate) and deliver intervention based on the hot cross bun and ii) topic-specific content e.g. discuss why exercise is important in COPD, discuss prevalence of anxiety and depression. To assess quality of delivery of each core task (section i) a 5-point Likert scale ranging from ‘not delivered at all’ to ‘delivered completely’ was agreed. As content (section ii) could only be considered as delivered or not delivered this was rated on a 0–1 scale. The treatment adherence scale was then presented to the process evaluation team for consideration of wording and understanding of items. It was iteratively piloted by the fidelity team on approximately 10 h of recordings until a workable and consistent treatment adherence scale was produced. One key change was the simplification of the 5-point quality scale for core tasks to a 3-point scale due to complexity in rating the 5 point scale.

##### Final TANDEM treatment delivery fidelity tool

The final method to assess treatment delivery comprised an initial section on therapeutic competence (as measured by the CFARS scale) and the specific treatment adherence scale with measurement of core tasks on a 1–3 scale (1 = not at all, 2 = partial, 3 = complete) and measurement of topic-specific content on a 1 = delivered, 0 = not delivered scale.

##### Analysis of the final TANDEM treatment delivery fidelity tool

It is intended that therapeutic competence will be scored by using the total competence scores from CFARS. Therapeutic competence will be analysed for total item mean and standard deviations, as well as median and interquartile range on each item of the CFARS. The proportion of Facilitators achieving fidelity (a score of > 30) across all of the ten items can also be reported. Treatment specific adherence will be scored separately from therapeutic competence. For each core task we will report the percentage of cases achieving high fidelity (> 80%). For topic specific adherence it is necessary to account for tailoring in scoring. Therefore, where items are not applicable (e.g., acceptance not discussed if a participant had good acceptance of their COPD) these will be omitted and the final score adjusted accordingly. The final score reflects the percentage of tailored content that should have been delivered that was delivered. The proportion of content that reaches greater than 80% fidelity is then calculated. A list of any ‘non TANDEM’ content delivered will also recorded.

Following piloting a fidelity handbook (available from the authors upon request) was produced with guidance on how each aspect of fidelity would be evaluated and specific detail on coding of the intervention specific treatment adherence scale was provided.

#### Treatment receipt

To measure treatment receipt, the number of sessions (and topics) each participant received, and all intervention materials provided, such as TANDEM handouts and BLF leaflets, was recorded. The minimum specified dose of the intervention was receipt of at least two CBA sessions and the handout on mood and COPD.

##### *Interviews*

Further information on treatment receipt was examined in qualitative interviews with Facilitators and patients. These were conducted as part of the process evaluation [21]. Patients were purposively sampled to reflect individuals in the TANDEM intervention arm who had completed versus dropped out of the CBA sessions, and attended versus not attended PR sessions. We aimed for a target of five participants per cell. Interviews were conducted after the 6 month assessment and were in person or telephone based on participant preference. The topic guide focussed on current experience of COPD/breathlessness, experience of being in the TANDEM study, relationship and working with the TANDEM Facilitator, experience of attending PR, suggested improvements to the TANDEM experience, perspectives on receiving TANDEM as part of routine care.

All facilitators were invited to participate in an individual interview with a target sample of fourteen interviews sought from different professional groups where possible. The number of patients seen by Facilitators (1–4; 5–8; 9 +) was also a factor in sampling. The main topics were training sessions, CBA sessions with patients, supervision, professional identity, perspectives on post-trial implementation.

#### Treatment enactment

No direct fidelity measure of treatment enactment and whether patients used the skills delivered was possible, however changes in key measures such as attendance at pulmonary rehabilitation and social outcomes were considered proxy measures of treatment enactment. In addition, qualitative interviews (as outlined above) with patients explored whether patients were enacting the skills learnt in the intervention.

## Results

### Design

The strategies used to enhance fidelity to the intervention are shown in Fig. 2 above.

### Training

#### Hiring Facilitators

**Fig. 3:**
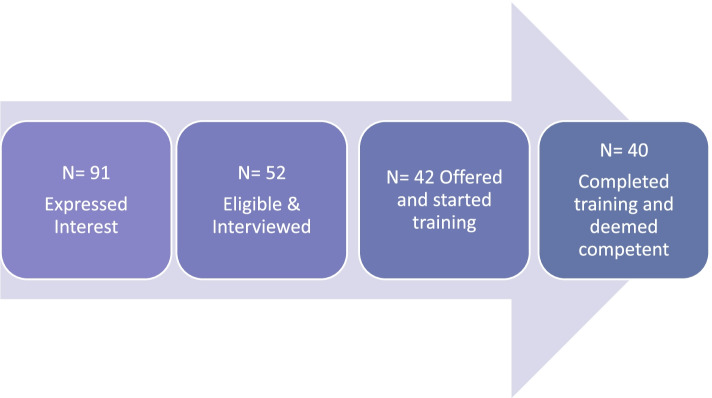
Flow of Facilitators Throughout Recruitment

Recruitment of Facilitators to the internal pilot and main trial continued for a period of 29 months from June 2017 to November 2019 (including a gap of seven months (December 2017 to June 2018) following the internal pilot). Figure 3 shows the recruitment process and Facilitator flow throughout. The most significant attrition was between initial expression of interest and interview (43%). This was mainly attributable to applicants providing insufficient detail, not meeting eligibility criteria, or changing their mind upon finding out more about the role. Of those interviewed (*n* = 52) approximately 81% were offered and took up training (*n* = 42). All but one individual (who had a change in job) offered initial training completed all three days and undertook standardized role play assessment of therapeutic competence. Overall, seven training programmes were delivered with a mean group size of five. Where the number of trainees in a group was below four the group was supplemented with non-tandem trainees (for example researchers working on behavioural science projects but not TANDEM) whose presence was purely to increase group size and ensure the group learning approach was maintained.

#### Training facilitators

Thirty-eight of the 42 participants who completed initial training and underwent standardized patient role play assessment met the therapeutic competence threshold of 27 on the first assessment. Of the four who didn’t achieve competence one declined the offer of further training, whilst three underwent additional training, two were subsequently deemed competent whilst one was deemed not competent and could not become a TANDEM Facilitator. Mean competence scores post training for those included as TANDEM Facilitators was 32.12 (range 27–41). The competencies that scored most highly were ‘interpersonal skills’ (3.84) and ‘collaborative relationship’ (3.83) with ‘guided discovery’ (3.32) and 'closure’ (3.39) scoring lower.

## Discussion

This paper describes a comprehensive strategy for the enhancement and assessment of fidelity in a complex intervention (TANDEM), following guidelines recommended by Toomey et al. [26]. We describe in detail the development and planned approach across all five fidelity domains as described by the NIH BCC [27, 28]. Assessment of all domains is rare [39] and we hope our description will be of benefit to others when aiming to design and report comprehensively on fidelity. Further, by detailing our strategy for assessment of treatment delivery, receipt, and enactment prior to data analysis we aim to increase transparency and reduce risk of bias in interpretation of results.

We hypothesise that our enhancement strategies will be important to ensuring delivery of TANDEM with fidelity. In particular, initial training was standardised, and Facilitators were provided with comprehensive support with manuals, participant materials and ongoing supervision where further training needs are addressed. The importance of supervision post-training has been reported previously [40–42] and may be particularly important for novice CBA therapists such as those in TANDEM [43]. Supervision may also be important to reduce therapeutic drift [44]. The extent of therapeutic drift will be measured in the current study by assessing fidelity of treatment delivery longitudinally for Facilitators with caseloads of more than ten participants.

We also report a structured approach to recruitment of Facilitators where only individuals who fulfil initial criteria and pass a competence assessment following completion of training were invited to be TANDEM Facilitators. It was hypothesised that this approach may support fidelity as individuals had high levels of motivation and commitment. Whether our approach to recruitment proves to be successful will be important for the implementation of the intervention if shown to be effective.

There was considerable learning in the development of the intervention specific treatment adherence scale. To aid other researchers we have highlighted issues that we discussed in detail in Table 1, as these considerations are likely to be pertinent to development of other interventions and fidelity tools. Of importance, was specifying the expected standard of care to be delivered by Facilitators. This enables consistency between coders and sets a specified standard below which Facilitators may be considered for needing further training.

**Table 1:** Points to consider when developing an intervention specific treatment adherence scale

Consideration	Implication and response
Unclear expectations around standard of care to be delivered?	This may risk inconsistency in coding. Clear expectations should be set which will also enable the Facilitator to be recommended to have further supervision or training if needed
Topic delivered in a different session to that planned?	If this is acceptable within a tailored intervention coding ‘full’ intervention sets rather than individual sessions may be helpful. Complementing assessment with data from additional data sources such as clinical report forms may also be helpful
Topic delivered out of prescribed order?	Separate coding for presence and delivery in the correct format may be necessary
Intervention strategies are dependent on individual (tailored)	If tailored a drop-down list of strategies may be necessary
Not all topics are appropriate in all contexts?	Delivery criteria may need to reflect if topics are not applicable and account for this in scoring
Is it obvious what is encompassed at each level of quality coding?	Descriptions may need to be provided for each quality level. Fewer levels e.g., three versus five may be preferable
Could non-intervention strategies be delivered and would this matter?	Additions to designed intervention strategies should be coded as well as omissions. These may be either beneficial or detrimental to the intervention

There was also considerable debate around whether it is preferable to randomly code individual sessions or full case delivery. If resources are limited the prior approach may allow for a greater number overall of participants to have sessions coded, which may be helpful if the fidelity tool is to be used for predicting outcome data. This approach may also have the benefit of greater ability to identify within provider variance [45, 46]. Facilitators were encouraged to tailor sessions to individual need and assessment of random sessions risks presenting an inaccurate assessment of adherence to content as a task not completed (for good reason) in a designated session may be addressed subsequently. This was a core consideration when we opted to code full intervention sets. The decision on which approach should be taken is likely to be informed by resources and also the particular needs of the fidelity assessment, which as argued previously should be described prior to analysis to minimise bias.

In the development of the fidelity process we were cognisant that audio-recording could feel threatening to potential Facilitators and we therefore took care to frame this as a non-judgmental process examining actions in a real-life context. In addition, all training was video-recorded with an emphasis on examining the fidelity of trainers delivery to illustrate integration of fidelity assessment at all levels of the study.

When developing our adherence scale, we identified the recognised tension between fidelity to, and adaptation of, the intervention. The TANDEM intervention was explicitly designed with tailoring embedded, as it is generally agreed that tailored interventions are likely to be more effective than one size fits all [47]. To overcome this tension when analysing fidelity both competence and adherence scorings included rating the appropriateness of the intervention delivered (with data gained from clinical report forms) with adjustment of scores depending on whether a task was omitted or added. Related to adaptability is the question of whether fidelity assessments should have a formative role by feeding back on skills and supporting the Facilitators’ future development. The importance of such feedback loops has been discussed by others previously [48]. Apart from in the training sessions, this was not done in TANDEM as it was anticipated that supervision would focus on skill development and whilst potentially helpful, we did not have resources to formally support this activity. We did not however preclude Facilitators using audio-recordings with their supervisors, and whether this occurred was monitored throughout the trial.

### Strengths and limitations

A particular strength of the TANDEM study was the approach of training multiple Facilitators to deliver the intervention across England. Assessment of fidelity in this study is therefore likely to be more generalisable to implementation in routine care than if only one individual had delivered all the intervention.

The methods we used are largely in line with the criteria for high quality in development of fidelity assessment strategies [39]. Some weaknesses in the strategy are however apparent, particularly with regard to the independence of coders for treatment delivery. Whilst one coder was independent of the study team, insufficient resources were available to have a second independent coder. The second coder (LS) was therefore a member of the intervention development team which may introduce bias, though this had the advantage that they were very familiar with the intervention. To minimise this bias the second coder maintained reflexivity and their assessments were used as quality checks with the primary coder providing all final scores.

A further potential weakness in our fidelity strategy was lack of direct measures of intervention receipt and enactment. Whilst this is explored in interviews and indicated through process measures in the trial (including social engagement and functioning and attendance at pulmonary rehabilitation) these are less robust than (for example) observation of participants enacting skills in their everyday life. Resources, however, did not extend to this.

We also note that domains of fidelity frequently over-lap. For example, supervision which ensures fidelity to treatment delivery also addresses training needs, similarly markers of treatment delivery such as home practice also relate to treatment enactment. This reflects the complexity of fidelity and the need to recognise that fidelity may be more helpfully considered a process as indicated in Fig. 1.

Finally, although we have aimed to provide a clear approach for scoring of fidelity and how we propose to analyse data we recognise that this is complex, particularly with a tailored intervention. Further testing of whether scoring is robust and can be helpful in explaining outcomes of study trials is therefore likely to be necessary.

## Conclusion

A strategy for enhancement and assessment of fidelity in the TANDEM trial has been presented and follows current best guidance. We have demonstrated fidelity at the training stage of TANDEM. Our intervention-specific adherence scale will enable us to report comprehensively on treatment delivery. A number of lessons have been shared. By presenting our strategy prior to analysis we also aim to minimise bias in assessment and allow for a transparent interpretation of results.

## Supplementary Information

**Additional file 1.**

**Additional file 2.**

## Data Availability

Data generated and analysed during this study is included in this published article. Study manuals and training materials are copyrighted but will be available upon reasonable request to the authors.

## Abbreviations

BLF: British Lung Foundation
CBA: Cognitive Behavioural Approach
CBT: Cognitive Behaviour Therapy
CFARS: Cognitive First Aid Rating Scale
COPD: Chronic Obstructive Pulmonary Disease
CTS-R: Cognitive Therapy Scale – Revised
NHS: National Health Service
NIH BCC: National Institute for Health Behaviour Change Consortium
PR: Pulmonary Rehabilitation
TANDEM: Tailored intervention for ANxiety and DEpression Management in COPD
