# Supplementary material for: Enhancing and assessing fidelity in the TANDEM (Tailored intervention for ANxiety and DEpression Management in COPD) trial: development of methods and recommendations for research design
Source: BMC Med Res Methodol. 2022 Jun 6;22:163. doi: 10.1186/s12874-022-01642-5 (PMC9171991; doi:10.1186/s12874-022-01642-5)
Supplement: Supplementary file 1 — Additional file 1. [file 12874_2022_1642_MOESM1_ESM.docx]

| **Strategies to enhance treatment fidelity as recommended by Borelli 2011 (**[**20**](#_ENREF_23)**)** | **Strategies used in TANDEM to enhance treatment fidelity** |
| --- | --- |
| **Design** |  |
| - Explicitly identify and use a theoretical model as a basis for the intervention, and ensure that the intervention components and measures are reflective of underlying theory. Use a protocol review group. | - TANDEM is based on clear theory (Cognitive Behaviour Therapy, Self-Regulation Theory, Self-Management) and is applied throughout sessions (17). Process evaluation team reviewed protocol. |
| - Pilot test the intervention and use feedback from participants and providers to refine adherence to the theoretical model and improve acceptability, feasibility, and potential effectiveness of the intervention. | - Intervention tested with expert group, pre-pilot and internal pilot with refinements at each stage to optimize acceptability, feasibility and effectiveness. |
| - Determine *a priori* the number, length, and frequency of contacts, and develop a monitoring plan to maintain consistency in dose. | - 6-8 sessions of 30-40 min duration held weekly, with documentation of each session delivered, and length, immediately post-session. |
| - Develop a plan for how adherence to the protocol will be monitored (audiotaping, videotaping). Monitor both intervention delivery and assessment administration (to ensure consistency of measurement). | - All sessions audiotaped. Random selection of cases reviewed by independent coder. Clinical report forms of session delivery and resources provided. |
| - Develop a plan to record protocol deviations (dose, treatment content) across all conditions and method of providing timely feedback to providers. | - Documentation of any protocol deviations in clinical report forms |
| - Develop a user-friendly scripted curriculum or treatment manual (print or via computer/handheld device) to ensure consistency of delivery and adherence to active ingredients of the treatment. | - TANDEM Facilitator Manual and crib cards provided to all facilitators to ensure consistency of delivery. Patient self-completion handouts for reinforcement of session content |
| - Plan for implementation setbacks (e.g., attrition of treatment providers). Videotape the trainings to ensure consistency for future trainings. | - Multiple trainings planned in case of provider attrition, trainings video-recoded and online version developed in case of necessity |
| **Training** |  |
| - Standardize training: Use the same trainers over time, use certified trainers, train all providers together, use standardized training materials, use video or audio tapes of expert delivery, develop a manual of training procedures and videotape trainings in case of provider attrition and need for future trainings. | - Training provided by same training team across all trainees, standard training materials. Training manual to support consistency across training sessions. |
| - Accommodate learner differences: Design training for diverse learning styles, train providers to deal with different types of participants, consider more intensive training and follow-up for less experienced providers. | - Visual Auditory Reading Kinesthetic approach used to accommodate learner differences. |
| - Assess skill acquisition: Use role plays with standardized patients followed by feedback to provider, score provider adherence to both intervention content and process using validated performance criteria, have a written exam pre and post training, develop criteria for initial certification. | - Post training assessment with a videoed role play with simulated patient, rated for minimum level of competency plus written evaluation |
| - Prevent skills drift: Booster sessions, patient exit interviews, periodic re- certification, audio or video record all encounters and code for treatment adherence, provide timely feedback, monitor patient drop-out rates of each provider. | - Frequent supervision, by accredited CBT practitioners. If concerns with regard to skills drift, raised with training team and booster training sessions provided. |
| - Enhance buy-in from providers: Foster provider self-efficacy and perception of organizational support. Explain the study design and rationale, the principles of research, and why it is important to prevent contamination and omission or addition of components not specified by the intervention. | - Importance of adherence to study manual (additions or omissions) highlighted in training and monitored through audio-recording (random cases assessed). Contamination prevented through randomization strategy   Additional TANDEM elements   - Between session practice for initial training attendees. |
| **Treatment delivery** |  |
| - Create relationships with providers to increase their comfort for reporting deviations (collaborative vs. hierarchical integrity monitoring). | - Fortnightly phone calls with research manager to discuss any concerns with implementing protocols. Access to clinicians to provide supportive clinical environment as might be available in clinical practice. |
| - Use a scripted curriculum or treatment manual. | - A comprehensive manual outlining aims and exercises to conduct in each session provided at the start of training. |
| - Assess non-specific effects through multiple methods and on an ongoing basis (patient exit interview, audiotape and code sessions, monitor participant complaints, provide feedback to provider). | - Post-intervention patient interviews, documentation of patient feedback, audio-recording of sessions, monitoring log of delivery. |
| - Minimize differences within treatments and maximize differences between treatments: manuals, frequent supervision to catch mistakes early, limit contact between providers of different treatment conditions, monitor provider expectations about treatment. | - Within each treatment standardized materials to be used. In intervention group a manual and clear guidance on tailoring to individual provided. Fortnightly clinical supervision. |
| - Ensure adherence to the protocol (content, dose, and process): audio or videotaped encounters, provider self-monitoring and patient exit interviews. | - Supervision to ensure adherence, audio recording all sessions, crib cards with tick boxes for facilitator self-monitoring. |
| - Check for errors of commission and omission, degree to which treatment components were delivered, and non-specific factors. | - Random selection of audio-recordings coded for omission or additions. - Assessment of materials delivered from case report forms |
| - Establish minimum competency levels, below which providers are given remedial training (e.g., adherence to <=80% of the components). | - Required standard of 27 on CFARS scale, below this re-training required |
| - Coders should be independent of the study, and blind to treatment assignment, participant progress and outcomes, and provider identity. | - Coding by personal independent of intervention development and trial. |
| - Use an independent group to review taped sessions and guess the treatment condition. | - Not possible as no recordings of control group   Additional TANDEM elements   - Use of case report forms to document sessions and topics delivered |
| **Treatment Receipt** |  |
| - Administer pre-post tests of client knowledge. | - Pre-post assessment of illness perceptions and behavior |
| - Present material in engaging manner. | - All materials developed with patient provider input to ensure engaging |
| - Ensure that written materials have appropriate health literacy. | - All materials developed with patient provider input to ensure easy to understand. |
| - Materials should be culturally relevant in terms of surface structure (photos) and deep structure (deeper cultural values). | - Culture considered in development of materials, tailoring allows consideration of individuals own cultural beliefs |
| - Provider should repeat information using multiple formats (verbal, pictures, written) | - Information provided verbally in sessions supported with written and diagrammatic leaflets and delivery via DVD. |
| - Participant should be queried for their understanding of the material covered in the visit. | - Each session begins with review of what had been understood from previous session and success with home practice tasks. |
| - Patients should role play the skills and receive coaching and feedback. | - Behavioural practice and rehearsal with feedback used for skills based activities e.g. breathing techniques |
| - Assess patients’ confidence to apply the skills delivered. | - All goals/activities agreed evaluated on 1-10 scale for confidence to ensure appropriate target level set. |
| - Structure the intervention around achievement-based objectives | - All goals set around specific behavior e.g., complete activity, practice exercise, reviewed and feedback provided each session |
| - Collect and review self-monitoring data. | - Self-monitoring between sessions reviewed at beginning of each weekly session, recorded in clinical notes but due to confidentiality not available for fidelity assessment |
| - Schedule follow-up visits and telephone calls to check in on understanding of the skills learned in treatment and level of adherence to recommendations. | - Telephone contacts prior to pulmonary rehabilitation. Trial procedures to assess outcomes   Additional TANDEM elements   - Patient and Facilitator interviews |
| **Treatment Enactment** |  |
| - Direct observation, self-report, provider report | - Interviews with patients to explore enactment of skills used.   Additional TANDEM elements   - TANDEM Folder (incorporating all hand outs given to provide an ongoing individualised resources) - Summary letter to general practitoner to enable ongoing care - Liaison with pulmonary rehabilitation team to facilitate consistency in care |

Supplementary Table One: Strategies to Enhance Fidelity in Tandem
