# Supplementary material for: Enhancing and assessing fidelity in the TANDEM (Tailored intervention for ANxiety and DEpression Management in COPD) trial: development of methods and recommendations for research design
Source: BMC Med Res Methodol. 2022 Jun 6;22:163. doi: 10.1186/s12874-022-01642-5 (PMC9171991; doi:10.1186/s12874-022-01642-5)
Supplement: Supplementary file 2 — Additional file 2. [file 12874_2022_1642_MOESM2_ESM.doc]

Supplementary Table Two: TANDEM treatment delivery fidelity measure

The below table should only be completed alongside the TANDEM fidelity handbook. There may be some variance in order of topic delivery due to tailoring and hence the order presented is just for illustration.

Date: 🞏🞏/🞏🞏🞏/🞏🞏🞏🞏 (dd/mmm/yyyy)

| **Therapeutic Competence** | Session | | | | | | | | Score (0-6) Refer to fidelity manual | Comments |
| --- | --- | --- | --- | --- | --- | --- | --- | --- | --- | --- |
|  | 1 | 2 | 3 | 4 | 5 | 6 | 7 | 8 |  |  |
| 1 Focus/structure |  |  |  |  |  |  |  |  |  |  |
| 2 Pacing |  |  |  |  |  |  |  |  |  |  |
| 3 Chunking/feedback/ capsule summaries |  |  |  |  |  |  |  |  |  |  |
| 4 Integration CBT into own professional model |  |  |  |  |  |  |  |  |  |  |
| 5 Collaborative relationship |  |  |  |  |  |  |  |  |  |  |
| 6 Guided discovery |  |  |  |  |  |  |  |  |  |  |
| 7 Interpersonal effectiveness |  |  |  |  |  |  |  |  |  |  |
| 8 Eliciting key components of the model |  |  |  |  |  |  |  |  |  |  |
| 9 Application of appropriate change techniques |  |  |  |  |  |  |  |  |  |  |
| 10 Appropriate closure |  |  |  |  |  |  |  |  |  |  |
|  |  |  |  |  |  |  |  |  |  |  |

| **Core Adherence Tasks** | Session | | | | | | | | 1= not delivered  2= partial delivery  3=complete delivery | Comments |
| --- | --- | --- | --- | --- | --- | --- | --- | --- | --- | --- |
|  | 1 | 2 | 3 | 4 | 5 | 6 | 7 | 8 |  |  |
| Agenda Setting |  |  |  |  |  |  |  |  |  |  |
| PHQ-9 |  |  |  |  |  |  |  |  |  |  |
| GAD-7 |  |  |  |  |  |  |  |  |  |  |
| Feedback From Home Practice |  |  |  |  |  |  |  |  |  |  |
| Refers to Hot Cross Bun (HCB) |  |  |  |  |  |  |  |  |  |  |
| Intervention based on HCB |  |  |  |  |  |  |  |  |  |  |
| Discuss Future Home Practice |  |  |  |  |  |  |  |  |  |  |

| **Content Adherence**  T=Topic | 0=Not delivered 1=Delivered | Comments |
| --- | --- | --- |
| T1: Introduces Self |  |  |
| T1: Explains TANDEM Aims |  |  |
| T1: Explains TANDEM structure |  |  |
| T1: Discusses Confidentiality |  |  |
| T1: Confirm consent for Audio-recording |  |  |
| T2: Elicit illness beliefs |  |  |
| T2: Elicit treatment beliefs |  |  |
| T2: Discuss how exercise is important in COPD |  |  |
| T2: Discuss coping with COPD |  |  |
| T2: Present Scenarios |  |  |
| T2: Discuss importance of acceptance |  |  |
| T3: Ask about patients experience of breathlessness |  |  |
| T3: Complete and share Hot Cross Bun (HCB) for breathlessness |  |  |
| T3: Complete spider diagram of breathlessness |  |  |
| T3: Highlight physical and psychological causes of breathlessness |  |  |
| T3: Provide Breathing handout |  |  |
| T3: Facilitator demonstrate breathing techniques |  |  |
| T3: Patient practice breathing techniques |  |  |
| T3: Discuss home practice |  |  |
| T3: Arrange next appointment |  |  |
| T3: Non Tandem Content Delivered (Specify) |  |  |
| T4: Feedback on home practice |  |  |
| T4: Ask about mood |  |  |
| T4: Discuss prevalence of anxiety/depression |  |  |
| T4: Complete a basic HCB |  |  |
| T4: Share HCB/Vicious cycles with patient |  |  |
| T4: Discuss patients primary goal |  |  |
| T4: Provide handout ‘Mood & COPD’ |  |  |
| T4: Discuss home practice |  |  |
| T4: Discuss summary letter to GP |  |  |
| T4: Arrange next appointment |  |  |
| T4: Non Tandem Delivery (specify) |  |  |
| T5- part1: Feedback on home practice |  |  |
| T5- part 1: Provide psychoeducation |  |  |
| T5- part1: Share HCB with patient |  |  |
| T5- part 1: Provide intervention techniques (drop-down list - choose all that apply) | |  |
| **Drop-down list**  Clinical education  Alteration of medication  Presentation of HCB  Psychoeducation  Addressing illness/treatment beliefs  Demonstration of Breathing exercises  Monitoring Diary  Activity Diary  Thought Diary  Problem Solving  Goal Setting  Reinforcement  Planning & Pacing  Graded Practice  Downward Arrow  Thought Challenging  Pros & Cons  Coping Self-Talk  Positive Log  Worry Tree  Distraction  Mindfulness  Relaxation | |
| T5- part1: Provide handout ‘COPD and Anxiety’ |  |  |
| T5- part1: Discuss home practice |  |  |
| T5- part1: Arrange next appointment |  |  |
| T5- part1: Non Tandem Content Delivered (Specify) |  |  |
| T5- part 2: Provide intervention |  |  |
| T5- part 2: Discuss home practice |  |  |
| T5- part 2: Arrange next appointment |  |  |
| T5- part 2: Non Tandem Content Delivered (Specify) |  |  |
| T6- part1: Feedback on home practice |  |  |
| T6- part1: Provide psychoeducation |  |  |
| T6- part1: Share HCB with patient |  |  |
| T6- part1: Provide intervention |  |  |
| T6- part1: Provide handout ‘COPD and Depression’ |  |  |
| T6- part1: Discuss home practice |  |  |
| T6- part1: Arrange next appointment |  |  |
| T6- part1: Non Tandem Content Delivered (Specify) |  |  |
| T6- part 2: Provide intervention |  |  |
| T6- part 2: Discuss home practice |  |  |
| T6- part 2: Arrange next appointment |  |  |
| T6- part 2: Non Tandem Content Delivered (Specify) |  |  |
| T7: Feedback on home practice |  |  |
| T7: (Applying CBT to other areas) Identify other problem |  |  |
| T7: Elicit HCB |  |  |
| T7: Share HCB with patient |  |  |
| T7: Provide intervention |  |  |
| T7: Provide referral advice/referral as appropriate |  |  |
| T7: Discuss home practice |  |  |
| T7: Arrange next appointment |  |  |
| T7: Non Tandem Content Delivered (Specify) |  |  |
| T8: Feedback on home practice |  |  |
| T8: Introduce topic -Living with COPD day to day |  |  |
| T8: Pros and cons of problem based coping |  |  |
| T8: Pros and cons of avoidance based coping |  |  |
| T8: Identify a current problem |  |  |
| T8: Work through the stages of problem solving |  |  |
| T8: Set a SMART goal with patient |  |  |
| T8: Explain problem solving & goal setting process |  |  |
| T8: Discuss home practice (including DVD) |  |  |
| T8: Non Tandem Content Delivered (Specify) |  |  |
| T9: Feedback on home practice |  |  |
| T9: Introduce topic –Preparing for PR |  |  |
| T9: Elicit attitude to PR including thoughts, feelings, expectations |  |  |
| T9: Discuss what to expect from PR |  |  |
| T9: Show photobook |  |  |
| T9: Problem solve as necessary |  |  |
| T9: Discuss telephone support |  |  |
| T9: Discuss letter to GP and PR |  |  |
| T10: Summary of TANDEM and next Steps |  |  |
| T10: Non Tandem Content Delivered (Specify) |  |  |

NB. Topics 1-3 are typically delivered in session 1, topic 4 in session 2, topics 5 and/or 6 in middle

sessions, topic 8 in the penultimate session an topic 9 and 10 in the last session.

As the intervention is tailored however there may be some variance in order.

Please use the TANDEM fidelity handbook for further guidance

Additional comments:

Researcher Rated by

Full Name ______________________________

Signature _______________________________
